# Autonomous Robotic Feature-Based Freeform Fabrication Approach

**DOI:** 10.3390/ma15010247

**Published:** 2021-12-29

**Authors:** Xinyi Xiao, Hanbin Xiao

**Affiliations:** 1 Mechanical and Manufacturing Engineering Department, Miami University, Oxford, OH 45069, USA; xiaox8@miamioh.edu; 2 School of Transportation and Logistics Engineering, Wuhan University of Technology, Wuhan 430063, China

**Keywords:** robotic additive manufacturing, autonomous, collision-free

## Abstract

Robotic additive manufacturing (AM) has gained much attention for its continuous material deposition capability with continuously changeable building orientations, reducing support structure volume and post-processing complexity. However, the current robotic additive process heavily relies on manual geometric reasoning that identifies additive features, related building orientations, tool approach direction, trajectory generation, and sequencing all features in a non-collision manner. In addition, multi-directional material accumulation cannot ensure the nozzle always stays above the building geometry. Thus, the collision between these two becomes a significant issue that needs to be solved. Hence, the common use of a robotic additive is hindered by the lack of fully autonomous tools based on the abovementioned issues. We present a systematic approach to the robotic AM process that can automate the abovementioned planning procedures in the aspect of collision-free. Typically, input models to robotic AM have diverse information contents and data formats, hindering the feature recognition, extraction, and relations to the robotic motion. Our proposed method integrates the collision-avoidance condition to the model decomposition step. Therefore, the decomposed volumes can be associated with additional constraints, such as accessibility, connectivity, and trajectory planning. This generates an entire workspace for the robotic additive building platform, rotatability, and additive features to determine the entire sequence and avoid potential collisions. This approach classifies the uniqueness of autonomous manufacturing on the robotic AM system to build large and complex metal components that are non-achievable through traditional one-directional AM in a computationally effective manner. This approach also paves the path in constructing an in situ monitoring and closed-loop control on robotic AM to control and enhance the build quality of the robotic metal AM process.

## 1. Introduction

Robotic AM's continuous rotatability of the building geometry enlarges the design space and complexity compared with traditional metal AM or subtractive processes. The conventional AM process is constrained in the single material accumulation direction that creates support structures underneath the overhang surfaces, specifically for the metal AM process. These supports must be removed using extra post-processing steps, and meanwhile, it could lead to a larger thermal distortion on the overhanging volumes [1]. In contrast, robotic AM achieves the continuous change of building orientation during the material deposition process, transforming overhang surfaces into a support-free manner under a different orientation. Therefore, utilizing such robotic AM can significantly reduce the heavy amount of support structures. Furthermore, changing deposition directions frequently can also avoid thermal stress concentration. Gaining the abovementioned advantages from robotic AM, a few issues come up with utilizing such a process are discussed below:

1. Freeform fabrication on robotic AM is not constrained as a planar form. Since not all building features are below the deposition nozzle, the collision risk between the building volume and the machine increases while the complexity of the process increases.
2. The selection and determination of the additive features/volumes for the robotic AM heavily rely on human interpretation of the given 3D models.

Thus, the correctness of the volumetric decomposition into additive features and the determination of their orientations is urgently needed. Intuitively, enabling multiple orientations in a continuous deposition process mimics the Boolean operations of the 3D features in the computer aided design (CAD) feature-based modeling process. Therefore, such feature addition can change from the traditional one-directional build-up to multi-directional material deposition. 

**Figure 1:**
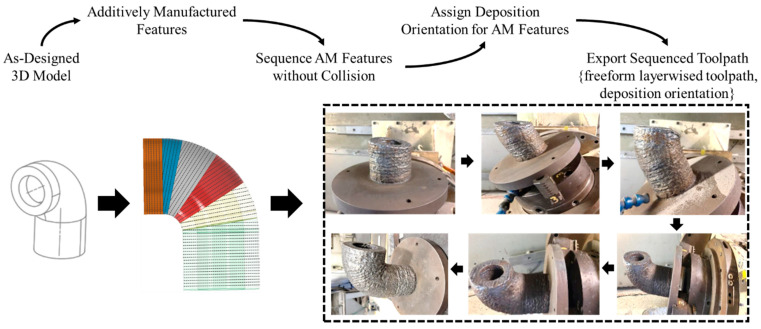
Overall workflow of robotic additive manufacturing.

In the current robotic additive workflow, manual separation of the input geometry into a few AM features requires expert knowledge, and a feasible manufacturing plan cannot always be achieved. Figure 1 provides an entire workflow of the robotic multi-axis additive process. In this workflow, planning the manufacturing process requires identification, sequence without collision, and trajectory generation of all AM features. Two types of collisions may happen in the AM: *local collision*—among the AM features and the robot, and *global collision*—between the building substrate and the robot. Currently, the process planning remains manual or semi-manual activities driven by expertise.

As shown in Figure 1, manual decomposition of the given geometry is the prerequisite to automating such a multi-variability process. However, due to the design complexity for additive manufacturing, the input components cannot be simply decomposed through planar cuts [2]. Moreover, the planar decomposition creates a positional multi-axis material deposition, the utilization of the robotic freeform additive feature cannot be fully explored. 

The major methods are concluded below for depositing materials through robotic additive manufacturing:

- Conformal freeform slicing;
- Adaptive slicing through the extracted medial axis.

These methods are capable of depositing material if a single feature is given to the system, such as an extrusion-based model, sweeping feature. However, a multi-feature complex model cannot be processed using the aforementioned two primary solutions. Therefore, the major task in designing a collision-free solution on a robotic AM system would require: (1) feature-based volumetric decomposition, (2) interaction among all decomposed AM features and the robot to ensure the collision-free processing condition.

Our approach to solving this problem involves the creation of valid convex-freeform surfaces from concave edges that can be applied to decompose the geometry. Nonetheless, not every concave edge loop on one geometry can be formed into a valid surface due to their shared surfaces with different curvatures. Our first proposed algorithm constructs a connectivity graph among all elements in a 3D model. Then, the attributes adjacency matrix is generated for grouping the concave edges within all elements. The convex-freeform shape surfaces can be generated from the grouped concave edges to ensure the zero possibility of local collision. The advantage of considering local collision into decomposing can be easily examined. After the decomposition, the only collision that needs to be considered in sequencing is the global collision. If the global collision is detected, it refers to the collide between the robotic deposition nozzle and the building substrate. Since constraining robot motion is a more complicated process and includes multiple decisions, the modification of the AM feature is a relatively straightforward adjustment. Our second algorithm will provide the modification method when a global collision exists. 

The main contribution proposed in this field is to provide a computerized non-collision process planning method of any given CAD model on a robotic AM system without human interpretation. It has significant benefits in saving manual work time and providing a process solution on a robotic additive manufacturing system. The problems that will be solved in this paper are stated as follows:

(1) Decompose any given geometry into feature-based additive volumes.
(2) Sequence all AM features without a collision. The as-built CAD model will be modified if a global collision is detected. The new sequence will be updated on the modified as-built CAD model.
(3) Two-dimensional planar and three-dimensional freeform slicing methods will be applied to the sequenced AM features for generating the overall trajectory on the geometry.

## 2. Literature Review

Building up a geometry on a robotic AM system can be categorized into two methods:

1. Decompose the geometry into features that need to be built along with the associated building orientation.
2. Generate a non-planar adaptive slicing method for the overall geometry, instead of flat layer slicing with uniform layer thickness.

This section will discuss recent manufacturing strategies—decomposition and non-planar adaptive slicing methods in the robotic additive system.

*Decomposition in Robotic AM:*

The significant problem among these methods lies in decomposing the geometry since sequencing and collision highly depend on the decomposition. Thus, providing an appropriate decomposition is the first and foremost step in autonomous robotic additive manufacturing. 

The major solutions for delivering such an autonomous manufacturing system are shown in Table 1. A shared among these studies is that they only focused on automating partial steps in the process planning. The deficiencies of these approaches have been exposed in Table 1.

With the development of CAD, much attention has been paid to the 3D model decomposition and feature recognition from different aspects. Sundaram and Choi [3] started to segment the 3D model and the building orientation for multi-axis deposition in the early stage. The remaining volume that cannot be built through the building orientation will be perpendicular to the previous orientation. This method will create more than necessary segmented volumes, and the collision cannot be avoided if the input geometry is complicated. To describe the input 3D model, Lee and others [5,6,7] extracted topological information, such as vertices, edges, and faces, to create feature information. However, the segmented elements have no manufacturing meanings, thus preventing this strategy from being applied to robotic additive manufacturing. Similarly, Jain et al. [9] developed a hierarchy graph to describe the shape information, but this cannot prevent any collision among decomposed features. In addition, Liu et al. [8] evaluated the paired points distance on the 3D models and classify the points using such distance. Still, this strategy cannot provide meaningful decomposed volume for multi-axis additive manufacturing. Luo et al. [13] and Chen et al. [14] developed methods for assembling components to form a complete 3D model. These strategies do not apply to the condition in which all decomposed elements are manufactured through one process. 

With the development of commercial multi-axis additive machines [24,25], Siemens NX [11] provides a semi-automated process planning for such operation, still requires lots of human interaction in decomposing the models to the suitable volumes of applying 2D planar or 3D conformal path trajectories. In addition to that, Rauch et al. [10] and Kapil et al. [12] used guided curves to specify the deposition orientation, but this process needs CAD expertise actions. Therefore, considering combining decomposition and multi-axis manufacturability, researchers developed a few automated process planning strategies [15,16,17,18] for depositing material along with multiple orientations in a support-less building manner. However, these approaches have the limitations that are concluded below:

(1) Xiao and Joshi [17], Wu et al. [15], and Murtezaoglu et al. [16] searched and created volumes that are allowable to be built support-less along with certain orientation. However, these process plans are primarily applied to positional multi-axis additive-rotatability for positioning the geometry. Therefore, freeform deposition cannot be fully exploited.
(2) Foskey and other researchers [18,19,20] utilized medial axis information to specify the building orientations. However, it cannot be applied to the symmetrical structures and other deficiencies as described in 1)
(3) Kapil et al. [23] calculated the deposition orientation through the series of parallel layers offsetting information. However, this strategy can only be applied to the geometries with constant one contour in the layers.
(4) Dai et al. [21] counted the available voxel through layer-wised projection to form the freeform layer. However, this strategy is constrained by a low print quality caused by an easy-skipping voxel deposition and a noncontinuous deposition path.

Table 2 visualizes the decomposition difference between the existing and proposed methods. 

The major constraints of applying the abovementioned methods are listed:

(1) The potential failure of decomposition will happen on the geometry in which one concave edge loop locates on multiple faces.
(2) This constrains the input can only be tree-shaped structures.
(3) Only planar or revolving cuts are used in decomposing the volume. Therefore, the robotic capability is not fully utilized.

*Slicing/Path Planning in Robotic AM:*

Instead of allowing motion in a translation movement along with x, y, z axes, the robotic additive allows more degrees of freeform. Such a process can be visualized by stacking multiple planar/freeform layers to create 3D geometries. Table 3 presents the current technologies that use a robotic system to produce 3D models. 

The initial advantage of robotic additive manufacturing is improving the surface finish on the curvature surfaces [42]. Due to the staircase effect along the slanted surfaces or curved walls, the planar layer deposition can be replaced and overcome using the freeform deposition along the curvature surfaces. Another limitation of the traditional layerwise deposition that can be overcome through the robotic additive system is the weak strength [33,37]. The conformal printing strategy can improve the material bonding along the curvatures. Thus, part strength can also be increased in specific directions. The path generation on a defined curvature layer has been widely studied by including the kinematics motion on the robots and acceleration and deceleration of the deposition motion [38,43,44,45]. These deposition path generation and robot trajectories computation strategies consider the behavior of robotics additive motion to prevent any collision between the freeform surface and the deposition nozzle. Other researcher [46,47] proposes the process map to optimize the build quality in terms of the process parameters, but not in the manufacturing deposition trajectory. However, all the above-mentioned techniques have limitations in building a 3D model that includes complex features. Thus, the feature-based deposition approach to overcome such limitations is urgently needed.

## 3. Methodology

### 3.1. Problem Statement and Solution Overview

**Figure 2:**
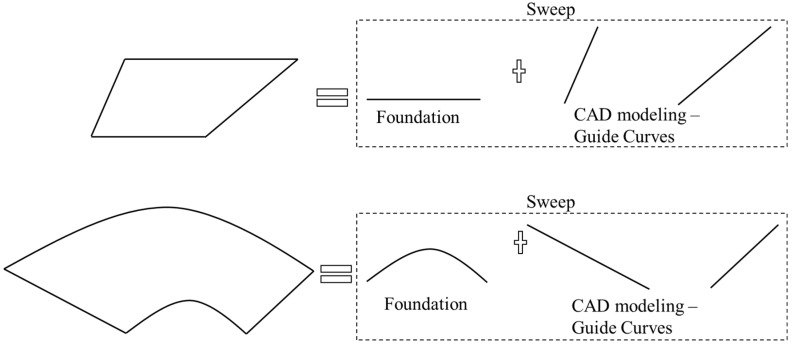
AM volume satisfying requirements in CAD modeling system.

The goal of decomposing the as-built 3D models is to reversely convert a multi-feature geometry to several single-body features. In Figure 2, we define the single-body feature as using one planar/freeform surface be the foundation and sweeping along one/multiple guide curves. By sweeping the foundation surface through the guide curves, the 3D feature can be modeled.

**Figure 3:**
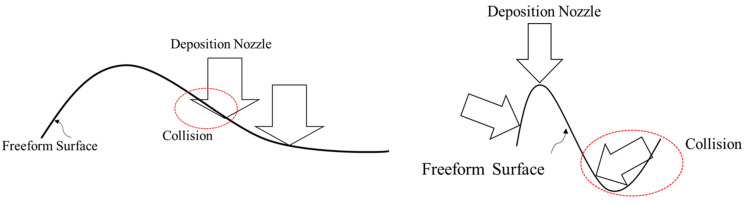
Local collision conditions.

However, only considering decomposing the model to several single-body features cannot ensure the non-local collision condition. Thus, an approach is proposed to ensure the decomposed feature can meet such requirements. Essentially, the local collision can happen between the AM feature and the robotic deposition nozzle because of the freeform layering. The creation of the freeform geometry would allow: (1) portion of the geometry higher than the robotic deposition nozzle along with the building orientation; (2) the oriented robotic nozzle interfere with the freeform layer concave region on the freeform geometry. Figure 3 presents the two types of local collisions discussed in this paper. 

The first type of collision can be avoided by modifying the orientation of the nozzle to be perpendicular to the freeform surface. However, the second type of collision may happen due to the varied machine tool size and the freeform surface topology. The local collision may occur at the concave zone on the surface.

In order to prevent such local collision problems and satisfy the decomposed volume modeling condition, the problem can be defined:

- $M=\sum {\mathrm{Sweep}\;\mathrm{Volume}}_{i}$;
- ${\mathrm{Sweep}\;\mathrm{Volume}}_{i}=\mathrm{sweep}\left({\mathrm{foundation}\;\mathrm{surface}}_{i},{\mathrm{guide}\;\mathrm{curves}}_{i}\right)$;
- ${\mathrm{foundation}\;\mathrm{surface}}_{i}=\mathrm{convex}$;
- ${\mathrm{Sweep}\;\mathrm{Volume}}_{i}\cap {\mathrm{Sweep}\;\mathrm{Volume}}_{j}={\mathrm{foundation}\;\mathrm{surface}}_{j}$;
- ${\mathrm{Sweep}\;\mathrm{Volume}}_{i}=\mathrm{conformal}\sum \left({\mathrm{foundation}\;\mathrm{surface}}_{i}\right)$.

**Figure 4:**
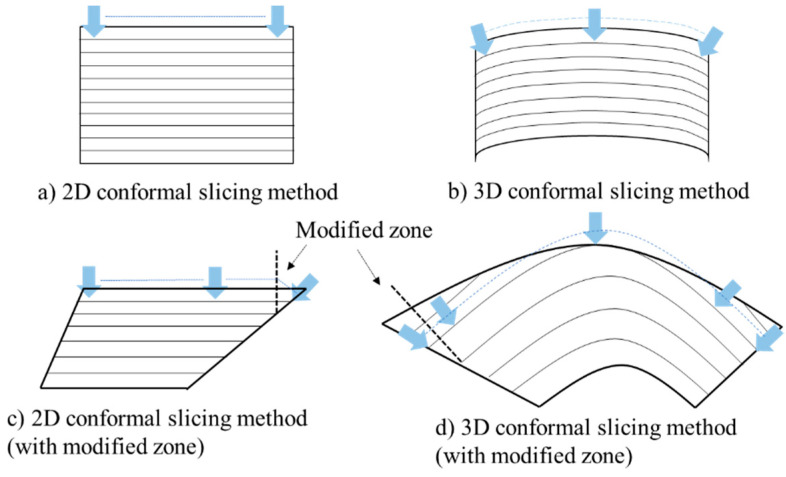
AM volume slicing strategies on a robotic system using adaptive conformal trajectory.

The concave edges exist in any model containing two or more CAD modeling features, and they can be considered the intersecting edges of the joining features. The formation of the decomposing surfaces (Figure 4) is unique and straightforward for fulfilling the following conditions:

- ${\mathrm{concave}}_{i}\in {\mathrm{face}}_{m,n},{\;\mathrm{when}\;\mathrm{face}}_{m},{\;\mathrm{face}}_{n}\;\mathrm{are}\;\mathrm{adjacent}\;|\;\mathrm{curvature}\left({\mathrm{face}}_{m}\right)=\;\mathrm{curvature}\left({\mathrm{face}}_{n}\right)$;
- ${\mathrm{surface}}_{i}=\mathrm{concvex}\;\mathrm{fill}\left({\mathrm{concave}}_{i}\right)$;
- $D_{i}=\mathrm{convex}\left(\sum {\mathrm{surface}}_{i}\right),{\;\mathrm{if}\;\mathrm{surface}}_{i}\;\mathrm{are}\;\mathrm{adjacent}$.

Through the decomposition, the individual AM volumes need to be built through 2D planar or 3D freeform conformal trajectory (shown in Figure 4). These conformal trajectories require adaptive modifications on the “overhanging” volumes by re-orienting the robotic deposition nozzle. This adaptive modification allows the overhanging regions to be built supportless.

From Figure 4, we can see that the overhang volume exists in both conditions. The traditional conformal trajectory uses parallelly slicing technique to build up the geometry and nearly does not allow any overhang volume. Instead, the overhang zone per layer utilizes the modified process parameters and the nozzle orientation to adjust the deposition volume. The modified orientation and the related process parameters will be discussed in the later section.

### 3.2. Proposed Approach

**Figure 5:**
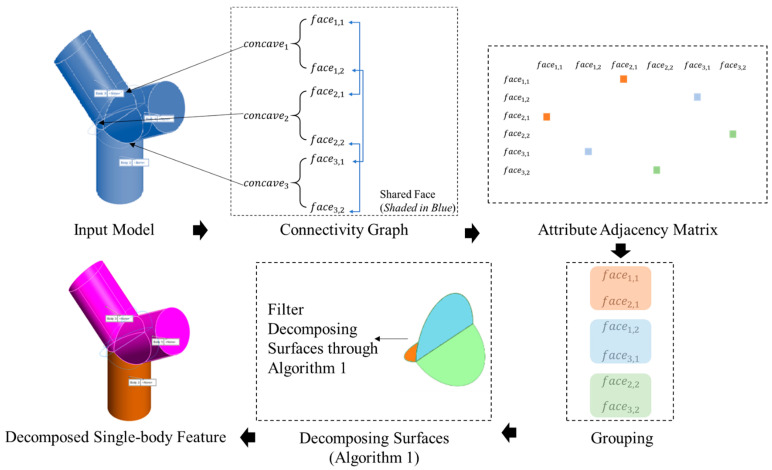
Overall workflow of a robotic AM single-body feature decomposition.

The prerequisites of processing decomposition for avoiding local collision are proposed in the previous section. This section comprises three algorithms used for decomposing, sequencing, and generating trajectories for a robot and segmenting the geometry into single-body features as the additive features. All the single-body features are local collision-free, satisfying the abovementioned conditions. The overview of Algorithm 1 from the input as-built CAD model to the decomposed single-body feature is presented in Figure 5.

After obtaining single-body features from Algorithm 1, Algorithm 2 can process all decomposed single-body features into a non-global collision sequence and provide conformal slicing orientation for all the decomposed features. If the global collision can be detected, the as-built CAD model will be modified to compensate for the collision risk. Later, algorithm 3 creates trajectory on AM features, including the robotic deposition nozzle approaching direction. All these volumes will be applied with adaptive conformal trajectories. If necessary, modifying the process parameters and the approaching orientation is needed.

#### 3.2.1. Algorithm 1: Decomposing into $V_{i}$

This section presents the autonomous decomposition algorithm for assigning the given as-built CAD model into single-body features built through the above-mentioned adaptive conformal trajectories.

**Figure 6:**
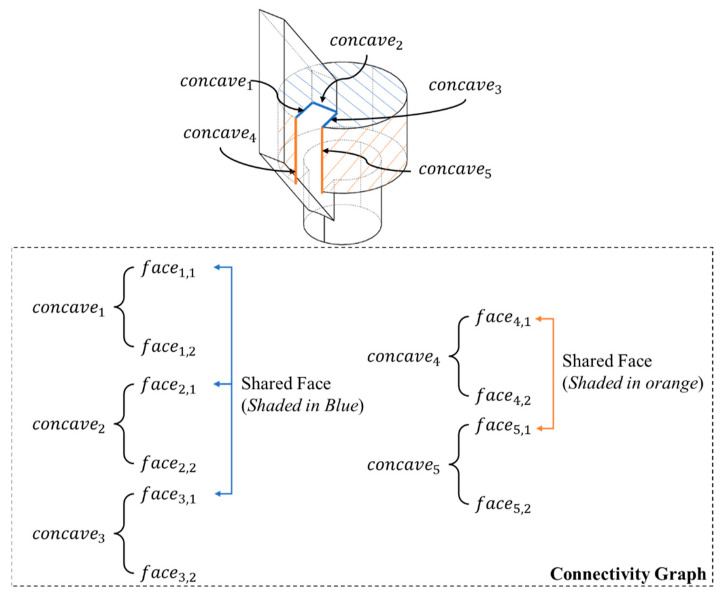
Connectivity graph generation by Algorithm 1.

**Figure 7:**
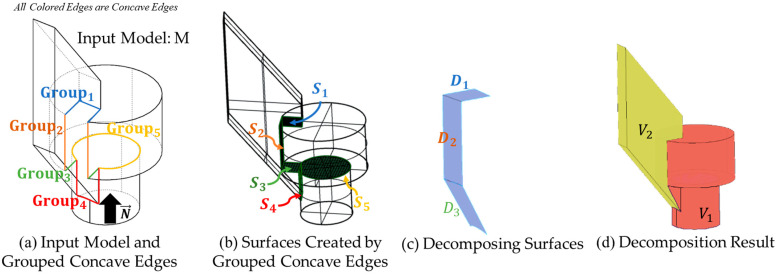
Visualized workflow of Algorithm 1.

An example has been processed through Algorithm 1 (Figure 6 and Figure 7) for illustrating the decomposition process. All colored edges shown in Figure 7a are the concave edges, and their corresponding color represents the group based on the connectivity graph and attributes adjacency matrix. From Algorithm 1, a connectivity graph can be computed for grouping the concave edges. In Figure 6, concave edges are first extracted, and their adjacent faces on the input model can be obtained as well. If a shared adjacent face of two or more concave edges, these concave edges will be grouped and form the attribute adjacency matrix.
**Algorithm 1:** Decomposition of as-built into single-body features. Autonomous creation of decomposed single-body features.Input: As-built 3D model M, initial building orientation vector ($\vec{N}$)Output: Decomposed volumes1.  Extract all ${\mathrm{edge}}_{k},{\;\mathrm{face}}_{l}$ on M
2.  Extract ${\mathrm{concave}}_{i}$, if $∡\left({\mathrm{face}}_{q},{\mathrm{face}}_{q+1}\right)\langle \pi \;||{\mathrm{face}}_{q}\cap {\mathrm{face}}_{q+1}={\mathrm{edge}}_{k}$
3.  **If** ${\mathrm{face}}_{l1}\cap {\mathrm{face}}_{l2}={\mathrm{concave}}_{i}$
4.   ${\mathrm{Connect}}_{i}$= {${\mathrm{face}}_{l1},{\mathrm{face}}_{l2}$}, Compute **Connectivity Graph** of $\mathrm{concave}\_{\mathrm{edge}}_{i}$
5.  **End**6.  **If** ∀Curvature(${\mathrm{Connect}}_{i}$) = ∀Curvature(${\mathrm{Connect}}_{j}$) || $\forall {\mathrm{Connect}}_{i}\cap \forall {\mathrm{Connect}}_{j}\neq \emptyset$
7.   ${\mathrm{group}}_{m}=\left\{{\mathrm{concave}}_{i},{\mathrm{concave}}_{j}\right\}$
8.   **Attribute adjacency matrix = [**${\mathrm{group}}_{m}$**]**9.  **End**10.  $S_{m}=\mathrm{convex}\_\mathrm{hull}\left({\mathrm{Attribute}\;\mathrm{adjacency}\;\mathrm{matrix}}_{m}\right)$, m∈[1,2,…,#group]
11.  **If**
$S_{x}\cap S_{x+1}\neq \emptyset$
12.  $Space_{x,x+1}=\left\{S_{x},S_{x+1}\right\}∋\vec{N}$
13.  $\theta_{x,x+1}\in Space_{x,x+1}||\theta_{x,x+1}=∡\left\{S_{x},S_{x+1}\right\}$
14.   **If** $\theta_{x,x+1}$ <π
15.    $D_{x}=S_{x}$
16.   **Elseif** $\theta_{x,x+1}>\pi$
17.    $D_{x}=\mathrm{convex}\_\mathrm{hull}(\cup \left\{S_{x},S_{x+1}\right\}∌\vec{N})$
18.   **End**19.  **End**20.  Decompose M by $\cup \;D_{x}$ into $\mathrm{single}-\mathrm{body}\;\mathrm{Features}\;(V_{y})$


In Figure 6, concave edges (1–3) share a face (shaded in blue) based on the connectivity graph. Therefore, these three concave edges are grouped. The same theory can be applied to concave edges (4–5). These two edges can then be grouped to be the same attribute. 

In Figure 7, the concave edges and the adjacent face curvature and connectivity group these edges are computed. Each grouped concave edge can then create the surfaces in b. If the subspace created by the adjacent surfaces (Figure 7b $S_{3},S_{4}$) includes the initial building orientation is convex, the new decomposing surface in Figure 7c $D_{3}$ will be created. The new decomposing surface is created by applying convex hull to the subspace that does not include the initial building orientation vector. The final decomposition result using these decomposing surfaces is shown in Figure 7d.

**Figure 8:**
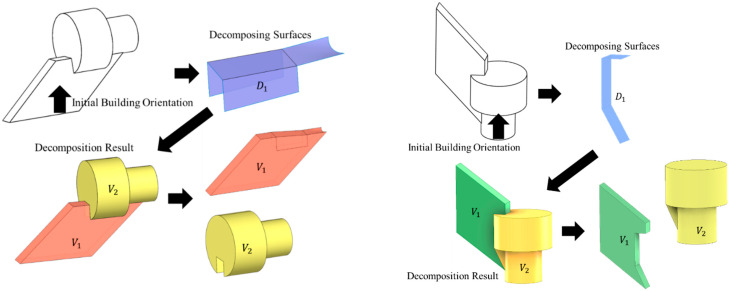
Decomposed single-body features of identical part by applying different initial orientation.

Since this decomposition algorithm is dependent on the initial building orientation, we present the different decomposition results by applying different initial orientations. All decomposed volumes ($V_{y}$) have a decomposing surface ($D_{x}$) that is convex-freeform that ensures the foundation surface on the decomposed feature is convex for avoiding local collision. The difference by applying different initial orientations to the identical model is shown in Figure 8.

#### 3.2.2. Algorithm 2: Sequencing and Modifying As-Built Model If Necessary

The local and global collision needs to be avoided in developing the autonomous robotic AM process. The local collision avoidance can be achieved through convex-freeform decomposition by Algorithm 1. The global collision will be detected through sequencing all single-body features with robotic motion into consideration. The detail of the algorithm is shown below. The as-built CAD model will be modified using the proposed algorithm if a global collision exists.
**Algorithm 2:** Sequencing Single-Body Features ($V_{y}$) and Modify As-built Model (M). Sequencing and modification of as-built model.
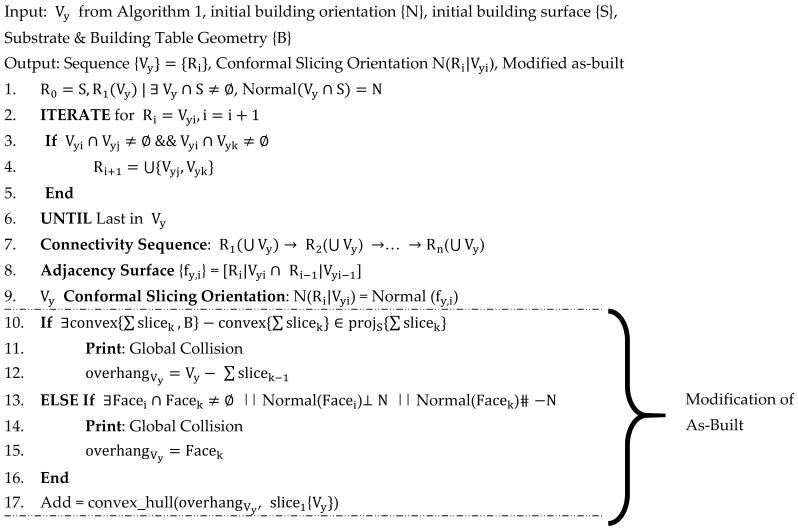


**Figure 9:**
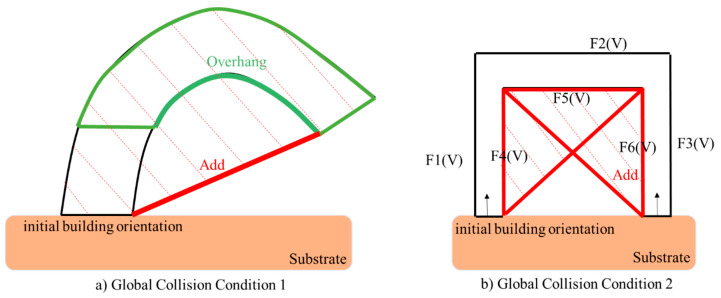
Two global collision conditions that can be detected and avoided by Algorithm 2.

Two examples of applying Algorithm 2 for detecting global collision are shown in Figure 9. In Figure 9a, due to the substrate and the robotic motion constraints, the nozzle (shown in grey) has no feasibility to achieve such overhanging surface area. Therefore, the global collision will happen here. Thus, the “Add” volume will be defined and applied to the as-built model. In Figure 9b, the nozzle has no access to the bridging geometry in the structure (we assume metal additive allows no bridging capabilities). This situation cannot be avoided due to the as-built model bridging structure. Thus, this situation is categorized into the global collision, which requires modification on the as-built model. The “Add” volume is calculated from Algorithm 2 and will be used to modify the as-built geometry for considering the building's feasibility.

#### 3.2.3. Algorithm 3: Trajectory Generation and Robotic Deposition Nozzle Approaches

In this section, Algorithm 3 will generate material deposition trajectories on the planar/freeform layers on the single-body features with adaptive modifications on the robotic deposition nozzle approaching orientations.
**Algorithm 3**: Trajectory Planning Generation. Trajectory generation.Input: $V_{y}$ from Algorithm 1 using updated As-built M (with Add) from Algorithm 2Output: Robotic AM trajectory, and approaching orientation1.   Extract $D_{x}$ from $V_{y}$ through Algorithm 12.   ${\mathrm{Slices}}_{V_{y}}$= offset($D_{x}$) $\cap V_{y}$, **Until** offset($D_{x}$) $\cap V_{y}=\emptyset$
3.  **If** ${\mathrm{proj}}_{{\mathrm{Slices}}_{V_{y-1}}}${${\mathrm{Slices}}_{V_{y}}$}=$\frac{{\mathrm{Slices}}_{V_{y-1}}\cdot {\mathrm{Slices}}_{V_{y}}}{{\left|{\mathrm{Slices}}_{V_{y-1}}\right|}^{2}}{\mathrm{Slices}}_{V_{y-1}}\neq {\mathrm{Slices}}_{V_{y-1}}$
4.   $\mathrm{modify}\_{\mathrm{zone}}_{{\mathrm{Slices}}_{V_{y}}}$= ${\mathrm{Slices}}_{V_{y}}-{\mathrm{proj}}_{{\mathrm{Slices}}_{V_{y}}}${${\mathrm{Slices}}_{V_{y-1}}$}5.   **For** $\forall \;P\;\mathrm{on}\;\mathrm{modify}\_{\mathrm{zone}}_{{\mathrm{Slices}}_{V_{y}}}$
6.     **Obtain** Q, Q$\in {\mathrm{Slices}}_{V_{y-1}}$||$\vec{PQ}⊥{\mathrm{Slices}}_{V_{y-1}}$
7.     **Approaching** = $\vec{PQ}$
8.   **End**9.  **ELSE If**
${\mathrm{proj}}_{{\mathrm{Slices}}_{V_{y-1}}}${${\mathrm{Slices}}_{V_{y}}$} = ∅
10.   **Approaching** = −Normal(P on ${\mathrm{Slices}}_{V_{y}}$)11.  **End**12.  **Export** ${\mathrm{Slices}}_{V_{y}}$ and Approaching

Algorithm 3 provides a strategy by orienting the nozzle direction from a perpendicular position to a tilted angle to fill the overhang volume.

## 4. Implementation and Result

Few examples are selected to demonstrate the effectiveness and efficiency of the proposed algorithms. One model would consume an experienced worker around 100 h without providing a feasible process planning to avoid collisions. The algorithms are coded into software by Rhino Grasshopper, C#, and Python running on an Intel i7-9750H CPU 2.60 GHz computer. All models cannot be autonomously manufactured without decomposing into buildable AM volumes or requiring extra attention in avoiding a collision. 

**Figure 10:**
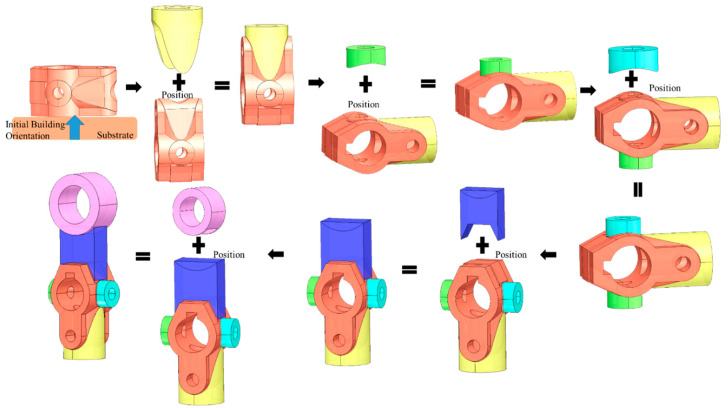
Process planning with first defined initial building orientation for model (a).

**Figure 11:**
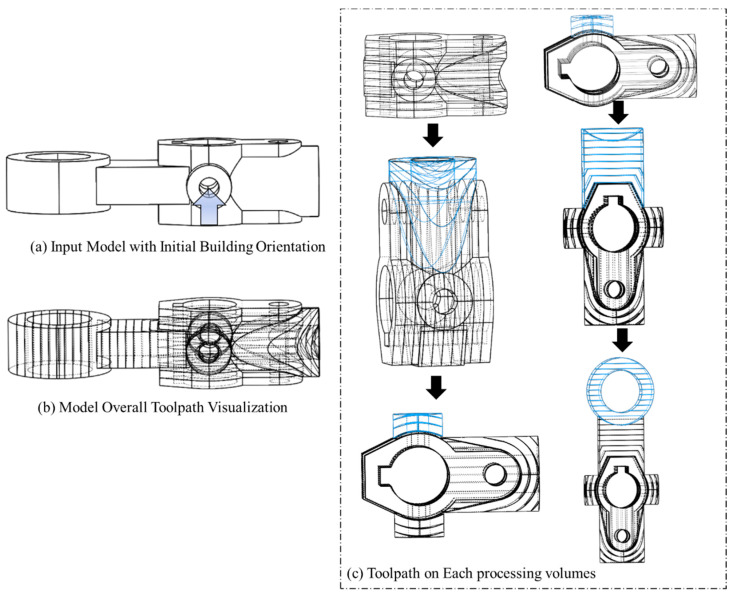
Trajectory accumulation on model with first defined initial building orientation for model (a).

**Figure 12:**
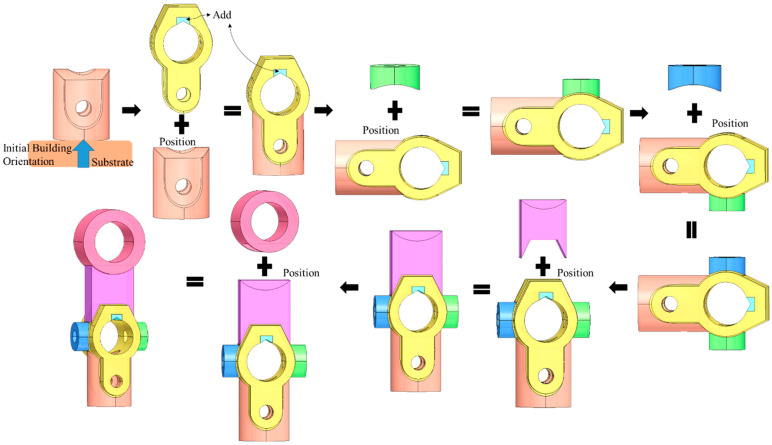
Process Planning with second defined initial building orientation for model (a).

**Figure 13:**
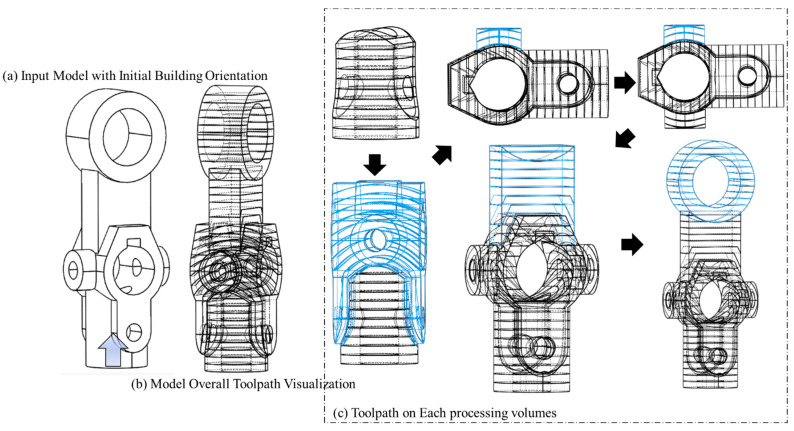
Trajectory accumulation on model with second defined initial building orientation for model (a).

Figure 10, Figure 11, Figure 12 and Figure 13 represent the processing results by providing the same as-built model (model (a)) with different initial building orientation and substrate size conditions. The decomposed single-body features with the manufacturing orientations and sequence are shown as well.

The below figure visualizes all the deposition trajectories on each single-body feature under the defined initial orientation.

The model is then processed with another selected initial orientation. Under this orientation, the Add volume is created through Algorithm 2 to prevent the global collision. The sequencing for processing single-body features is presented in Figure 12.

Similarly, all deposited features’ trajectories under the defined initial orientation are presented below.

By comparing Figure 10 and Figure 12, the manufacturing features and associated sequence also varied based on different initial building orientations. The modification of the as-built geometry for avoiding the global collision condition is also dependent on the selection of the initial orientation. 

**Figure 14:**
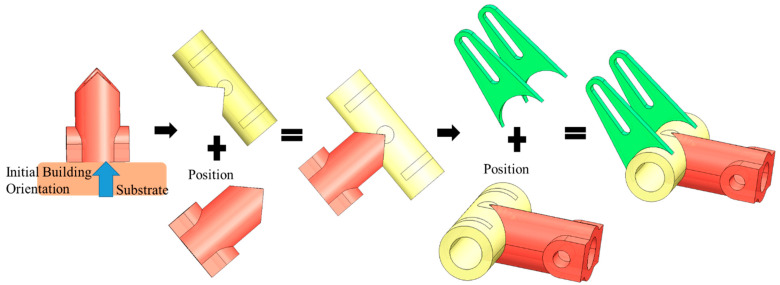
Process Planning with first defined initial building orientation for model (b).

Another model (b) (Figure 14 is processed through the proposed method that uses a robotic additive. The decomposed features are correctly assigned with the feasible processing sequencing without collisions are shown in Figure 14. 

**Figure 15:**
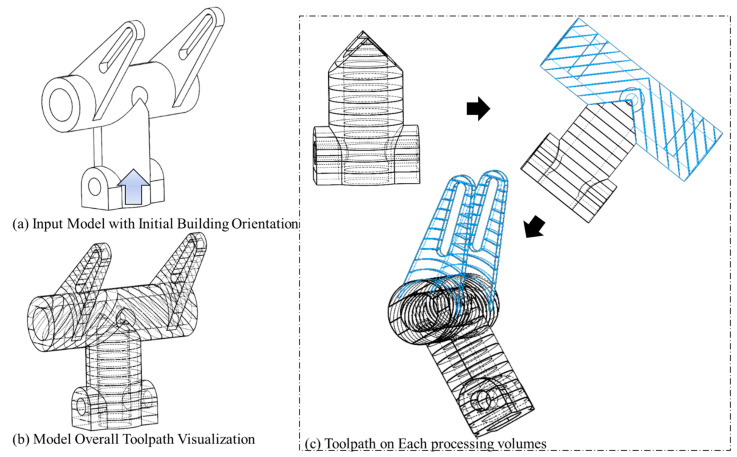
Trajectory accumulation on model with first defined initial building orientation for model (b).

The accumulated trajectories for this geometry are shown in Figure 15.

**Figure 16:**
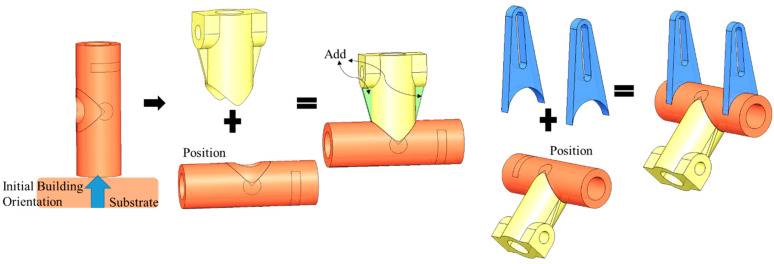
Process Planning with second defined initial building orientation for model (b).

This model is then processed with another initial orientation which is shown in Figure 16. Under this orientation, the Add volumes (green volumes) are created through Algorithm 2 to prevent the global collision. 

**Figure 17:**
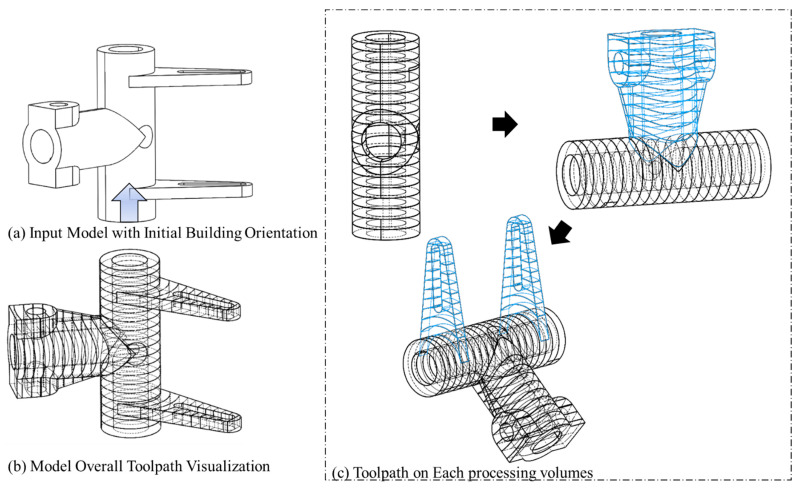
Trajectory accumulation on model with second defined initial building orientation for model (b).

The accumulated trajectories for this geometry, including the added volumes, are shown in Figure 17.

**Figure 18:**
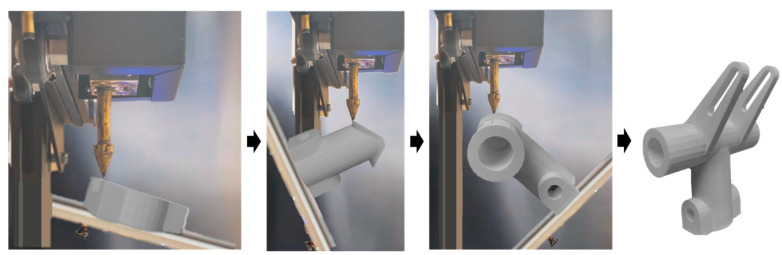
Printing Process for the model (b) in Figure 14.

Model (b) has been processed using the manufacturing strategy indicated in Figure 14 and Figure 15. The machine that has been used in implementation is a fused deposition machine with a modified continuously rotary building platform to fulfill the robotic multi-axis material deposition capability. The overall printing process is presented in Figure 18. In addition, model (b) has been continuously oriented to sustain each single-body feature's supportless deposition manner.

The success of model (b) indicates the feasibility of the proposed autonomous manufacturing strategy on the robotic additive system, specifically for the metal additive process. The metal additive process, including the directed energy deposition and wire arc additive, has a small supportable overhang angle. Therefore, the supportless manner using the proposed robotic manufacturing method overcomes the metal AM manufacturability limitation without any extra human interpretation on the geometries.

## 5. Discussion and Conclusions

An autonomous feature-based manufacturing strategy is proposed, which utilizes robotic freeform fabrication. Autonomous deposition and accumulation on a freeform layer and continuously changing the building orientations in one process overcomes the limits in design, manufacturing, and human interpretation on the geometries. Current techniques of a multi-orientation additive process require heavy human intervention in specifying the additive features with orientations and trial and error in collision avoidance. The proposed method efficiently computes the single-body features for a given 3D model such that the part can be fabricated under the Boolean combination of these features on a robotic additive system in a collision-free and supportless manner. The proposed algorithms have been verified by simulating the multi-orientation freeform layer generations and the experimental implementations. 

Future work will include the optimal computation on the process parameters for the metal robotic additive process to improve the quality and performance of the build. In addition, the proposed approach can be applied to Directed Energy Deposition (DED) with both powder and wire feed. Additional experiments using these are envisioned in the future to develop the proposed approach further.

## Figures and Tables

**Table 1:** Capability of existing decomposition methodology and corresponding deficiencies.

Varied Directional Deposition Strategy	Incapable
Projection-based Determination on the Manufacturable Volume [3,4]	Inefficient Decomposition; All decomposed volume only uses planar deposition Cannot fully exploit the robotic multi-axis capability Incapable of avoiding collision during manufacturing
Topological Feature Information [5,6,7] Pairwise Distance Evaluation [8] Shape Hierarchy Information [9]	Segmentation is not correlated with the manufacturing strategy Incapable of avoiding collision during manufacturing
Guided Curve Assisted Multi-Axis Deposition [10,11,12]	Human interpretation is still required in specifying the deposition orientation Decomposition the given geometry to a single feature still as a manual step
Chopper Partition [13,14]	Only applicable for creating individual pieces using assembly
Beam-Guided Search for Overhanging Volumes [15] Half-space Cutting Plane for Overhanging Volumes [16] Buildable Volume Determination Based on Manufacturability [17]	All decomposed volume only uses planar deposition; Cannot fully exploit the robotic multi-axis capability
Medial Axis Extraction and Volume Interpolation [18,19,20]	Incapable of handling symmetrical geometries; All decomposed volume only uses planar deposition; Cannot fully exploit the robotic multi-axis capability
Voxelization Freeform layer-wise deposition [21] Marching Cube decomposition [22]	Curved layers generation by counting shadowed voxels based on building accessibility, thus skipping voxel-sized deposition is normal Non-continuous path generation creating rough geometry finish
Adaptive Orientation using Offsetting Parallel Contours [23]	Only Applicable to the geometries that have a consistent number of parallel slicing contours

**Table 2:** Decomposition results from existing methods and proposed one.

	Ren et al. 2008 [26]	Liu et al. 2019 [27]	Xiao et al. [17]	Proposed Methodology
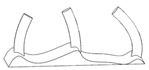	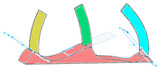	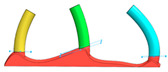	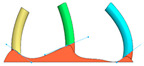	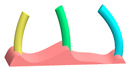
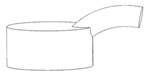	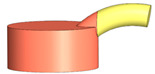	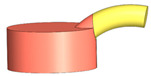	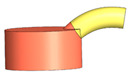	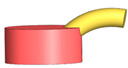
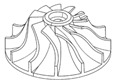	Not Applicable	Not Applicable	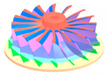	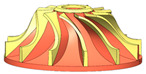
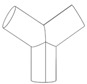	Not Applicable	Not Applicable	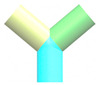	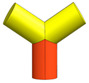

**Table 3:** Robotic additive slicing and path planning strategies overview.

Slicing/Path Planning Method	Result	Incapable
Adaptive slicing algorithms based on the cusp height and residual height [28]	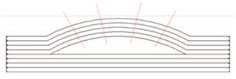	Non-collision detection; It cannot be applied to multi-feature geometry
2.5D layerwise path planning [29,30]	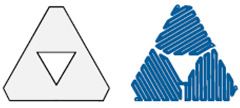	Planar-wised material accumulation
Planar surface additive strategy for productivity and geometric accuracy [31]	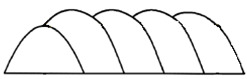	Planar-wised Solution
Freeform path planning on curvature surfaces [32,33,34]	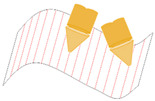	Single freeform layer deposition; Only for the coating material, not for 3D model deposition
Freeform weld path planning [35]	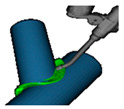	Not suitable for robotic additive 3D models
Spiral infill/climbing strategy [36]	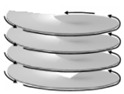	Only suitable for special geometric model
Conformal layer AM [37,38,39,40,41]	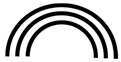	Geometric limitation; Volumetric decomposition is needed for complex models
